# A 10-year time-series analysis of respiratory and cardiovascular morbidity in Nicosia, Cyprus: the effect of short-term changes in air pollution and dust storms

**DOI:** 10.1186/1476-069X-7-39

**Published:** 2008-07-22

**Authors:** Nicos Middleton, Panayiotis Yiallouros, Savvas Kleanthous, Ourania Kolokotroni, Joel Schwartz, Douglas W Dockery, Phil Demokritou, Petros Koutrakis

**Affiliations:** 1 Department of Environmental Health, Exposure, Epidemiology & Risk Program, Harvard School of Public Health, 401 Park Drive, Boston, MA 02215, USA; 2 Cyprus International Institute for the Environment and Public Health in association with Harvard School Public Health, 5 Iroon Str, Nicosia 1105, Cyprus; 3 Air Quality Section, Department of Labour Inspection, Nicosia 1493, Cyprus

## Abstract

**Background:**

To date, a substantial body of research has shown adverse health effects of short-term changes in levels of air pollution. Such associations have not been investigated in smaller size cities in the Eastern Mediterranean. A particular feature in the region is dust blown from the Sahara a few times a year resulting in extreme PM_10 _concentrations. It is not entirely clear whether such natural phenomena pose the same risks.

**Methods:**

The effect of changes in daily levels of particulate matter (PM_10_) and ozone (O_3_) on hospitalization for all, cardiovascular and respiratory causes in the two hospitals in Nicosia during 1 January 1995 and 30 December 2004 was investigated using generalized additive Poisson models after controlling for long- and short-term patterns as well as for the effect of weather. Meteorological records were reviewed to identify dust-storm days and analyses were repeated to quantify their effect on cardio-respiratory morbidity.

**Results:**

For every 10 μg/m^3 ^increase in daily average PM_10 _concentrations, there was a 0.9% (95%CI: 0.6%, 1.2%) increase in all-cause and 1.2% (95%CI: -0.0%, 2.4%) increase in cardiovascular admissions. With respect to respiratory causes, an effect was observed only in the warm months. No lagged effects with levels of PM_10 _were observed. In contrast, positive associations with levels of ozone were only observed the two days prior to admission. These appeared stronger for cardiovascular causes and independent of the effect of PM. All-cause and cardiovascular admissions were 4.8% (95%CI: 0.7%, 9.0%) and 10.4% (95%CI: -4.7%, 27.9%) higher on dust storm days respectively. In both cases the magnitude of effect was comparable to that seen on the quartile of non-storm days with the highest levels of PM_10_.

**Conclusion:**

We observed an increased risk of hospitalization at elevated levels of particulate matter and ozone generally consistent with the magnitude seen across several European cities. We also observed an increased risk of hospitalization on dust storm days, particularly for cardiovascular causes. While inference from these associations is limited due to the small number of dust storm days in the study period, it would appear imperative to issue health warnings for these natural events, particularly directed towards vulnerable population groups.

## Background

In the last 20 years, evidence on adverse health effects – both increased hospitalization and mortality – of elevated ambient levels of air pollutants has been accumulating [1,2], more so recently with the use of meta-analyses of single-city time-series analyses [3,4] or multi-city studies [5,6]. With the major exception of the APHEA project, a multi-centre European study with a common protocol [7] in as many as 15 and 29 European cites in its phases I and II respectively [8-10], the majority of evidence comes from US cities e.g. the National Morbidity, Mortality and Air Pollution Study (NMMAPS) [11,12]. While large cities in the Eastern Mediterranean, such as Athens, Tel Aviv and Istanbul were considered in APHEA, associations have not been investigated in smaller size cities where socio-economic factors (such as driving patterns, time spent outdoors and access to health care) as well as climatic conditions might vary considerably. A particular feature of the Eastern Mediterranean is episodes of re-suspended wind blown dust from desert regions, raising particle concentrations a few times a year considerably above European guidelines [13]. It is not entirely clear whether high levels of particulate matter from such natural phenomena pose the same risks on cardiovascular and respiratory health as particles from anthropogenic sources. With PM_10 _concentrations comparable to the high levels seen in other much larger Southern European cities as well as frequently affected by dust storms, the capital of Cyprus, Nicosia (population approximately 270,000) offers an opportunity to address this issue. Using a time-series approach, this study investigates associations between daily levels of (a) particulate matter with aerodynamic diameter < 10 μm (PM_10_) on non-storm and dust storm days separately as well as (b) ozone (O_3_) on counts of hospital admissions for all, respiratory and cardiovascular causes during the 10-year period 1995–2004.

## Methods

### Data and data sources

Based on ICD code of diagnosis for inpatient admission, all cardiovascular (ICD10 codes I00–I52) and respiratory (ICD10 codes J00–J99) admissions between 1 January 1995 to 30 December 2004 (i.e. a total of 3,652 days) were obtained from the two public hospitals in Nicosia (i.e. Nicosia General and Makarios Hospital) with information on gender, age, date of admission and discharge as well as whether inpatient was resident in the district of Nicosia. The daily volume of all-cause admissions in the same period was obtained from the Cyprus Statistical Services, aggregated in 8 age- and sex-strata – males and females, aged 4 or less, 5–14, 15–64 and 65 or older. Hourly measurements of PM_10 _and O_3 _were available from two ambient air quality monitoring stations: (a) a local traffic-representative station located centrally at the Nicosia General Hospital and (b) a station reflecting background levels in the rural location of Ayia Marina, approximately 40 km from Nicosia. Continuous monitoring of coarse particles was performed with TEOM (Tapered Element Oscilling Micro-balance) instruments. Data from the rural station were available only from 1997 for ozone and 1999 for PM_10_, reducing the study period to 8 and 6 years respectively. Thus, the main analyses presented here were based on a single exposure series as recorded at the traffic-representative station. Completeness of the main data series of air pollutants was generally satisfactory and concurred with previous practice, including the protocol used in APHEA. Commonly, missing values are replaced using a weighted average of values from other monitoring stations on that day. With only one other station, located rurally and thus not necessarily representative of exposure in the capital, imputation of missing values was not considered not least because availability of data did not cover the full period of investigation and would replace only a small fraction of the missing values. The background station was mainly used to aid in the identification of dust-storm days, since due to its rural location it is not affected by local traffic pollution. Nevertheless, the effect on the observed estimates of using the background station to define exposure was considered in sensitivity analyses. Daily averages of air and dew point temperature, relative humidity, wind speed, precipitation and barometric pressure based on hourly measurements of Thermohygrographs (i.e. instantaneous values) were obtained from the Cyprus Meteorological Services.

### Identification of dust-storm days

Dust storm events affect Cyprus at least a few days a year resulting in extreme PM concentrations, which sometimes may persist for a few consecutive days. To construct a calendar of such events, an iterative approach was used whereby starting with a pool of days (N = 773) with at least 1 hourly measurement of PM_10 _higher than 150 μg/m^3 ^recorded at Nicosia Central or higher than 100 μg/m^3 ^at the rural station (in both cases, 2 standard deviations (SD) away from the mean of hourly values on the logarithmic scale), paper-form meteorological records from the main International Airport at Larnaca (50 km from Nicosia) were reviewed to identify those days when a meteorological observer (as part of their hour-by-hour coding of weather and visibility conditions) noted poor visibility due to "dust in suspension" at any part of a given day, which did not refer to hazy conditions or dust from local sources i.e. a result of re-suspension. Candidate days were then cross-checked against a number of data-based criteria to assess the extent to which levels of PM_10 _on those days where indeed uncharacteristic, including (i) daily average levels of PM_10 _higher than 2 SDs away from the mean as recorded at the rural station, not as prone to traffic pollution and/or (ii) levels of PM_10 _higher than 2 SDs away from the mean in the centre of Nicosia after excluding hours of pick traffic and/or (iii) days identified as outliers (2 SDs away from the predicted value) using a predictive model of levels of PM_10 _based on levels the previous day and adjusting for weather variables including temperature, barometric pressure, precipitation and wind speed. The data-based criteria were not used to identify dust-storm days *per se *but to correct the original calendar which was based only on meteorological observations (i.e. either confirm, invalidate or propose additional days) with the main aim of categorizing days into *confirmed *events, where the criteria were in agreement with the meteorological observation and *suspected *events, where all criteria suggest an event that might have been overlooked or coded otherwise by the observer. Possible markers of dust storm events used in the past, such as low carbon monoxide levels [14], reduced visibility range [15], or based on investigation of aerosol optical depth [16,17], were not electronically available. Finally, in accordance to previous practice [16,18-20], backwards wind trajectories for up to 4 days ending in Cyprus (35°N, 33°E) on the day and about the time the meteorological observation was made were used to track the possible source of each identified event in the Sahara or Arabian peninsula using the National Oceanic and Atmospheric Administration (NOAA) HYSPLIT model.

### Statistical analyses

Only days with at least 12 hourly measurements were used to calculate daily averages of air pollutants (PM_10 _and O_3_) as well as daily 8-hour maximum moving average for O_3_. The effect of changes in levels of air pollutants on the number of daily admissions was investigated in Poisson regression models (a) as linear terms, expressed as percentage increase in mean number of daily admissions per 10 μg/m^3 ^or 10 ppb increase in levels of PM_10 _and O_3 _respectively, (b) across quartiles of increasing levels of PM_10 _(after including and excluding dust storm days) and O_3 _to assess non-linearity of effects and (c) to include a categorical variable for dust storm days and restrict the estimation of a linear effect to non-storm days only (i.e. Dust Storm Day (= 0 or 1) + PM_10 _daily average × [1-Dust Storm Day]). The magnitude of effect on these days was then compared to the effects seen across quartiles of all non-storm days with increasing levels of PM_10_. In accordance to previous practice, to ensure that extreme values of PM_10 _(in this case, most likely to be a result of air blown dust) would not influence the estimation of linear effects, the main analyses excluded days with average PM_10 _concentrations greater than 150 μg/m^3 ^(25 days). Furthermore, to assess the extent to which associations persist at lower ambient levels of air pollution, analyses were repeated restricted to days with daily averages of PM_10 _less than 100 μg/m^3 ^or 75 μg/m^3 ^(the European standard at the time). Finally, to assess and correct for overdispersion, or extra-Poisson variation in the data, models were repeated using negative Binomial models (i.e. an overdispersed Poisson distribution). Model fit was assessed by inspection of the overdispersion parameter, the model deviance as well as patterns and magnitude of the residuals.

Generalized Additive Models (GAM) with natural splines were used to remove long-term seasonality (starting with the practical choice of 40 degrees of freedom to capture 4 seasons over the 10 year period) and penalized cubic splines to control for possible non-linear effects of the meteorological variables on the outcome (with a maximum of 5 degrees of freedom). In order to control for short-term patterns of admissions, day of the week was included in the models as a categorical variable. Before including the air pollution variables in the model, a base model was constructed to remove seasonality i.e. long-term trend and weekly patterns. Minimising the absolute value of the sum of the partial autocorrelation function was used to assess the appropriateness of the degree of smoothing. To avoid oversmoothing and, thus, eliminating patterns due to the exposure under study, windows below 2 months were generally not considered [21]. The weather variables as well as the appropriate lags for these to include in the models were then chosen by minimizing the Unbiased Risk Estimator (UBRE) and/or the Akaike's Information Criterion (AIC). All models were checked for remaining autocorrelation by examining plots of the partial autocorrelation function and, if necessary, sensitivity of the inferences was assessed in autoregressive Poisson models. The final model controlled for long- and short-term trend, temperature on the same day as well as the two previous days (lags 1 and 2) and relative humidity on the same day. Wind speed, precipitation and barometric pressure were not considered as confounders of the association between air pollutants and hospitalization. The effect of ozone was assessed before and after controlling for the effect of PM_10_.

Same-day and lagged exposure (up to 2 previous days) were considered. Due to lack of statistical power, distributed lag models were not considered [22]. Similarly, due to the small number of daily cause-specific admissions, analyses were only stratified by gender (all ages combined) or age (i.e. younger/older than 15 years of age). Models were repeated to include interaction terms between levels of pollutants and season to investigate evidence for effect modification either (a) during cold and warm months indexed by monthly average temperature levels or (b) on days when average daily temperature was higher or lower than 20 or 30°. Finally, where possible (i.e. cause-specific investigation), all analyses were repeated to exclude non-Nicosia residents since these may represent transfers from other hospitals and, as such, can dilute any effect since day of admission may not accurately reflect day of exposure. Data manipulation was performed in STATA SE 9.0. Analyses were performed using the MGCV package in the R software (R 2.2.0).

## Results

Admissions in Nicosia hospitals nearly doubled in the 10-year period. There has been a 3-fold increase in cardiovascular admissions rising from an average of 1 in the early years to 4 towards the end, averaging at 3 daily. For respiratory causes, admissions averaged approximately 4 daily in much of the 10 years. Table 1 shows summary statistics of the daily number of all-cause and cause-specific admissions. Numbers are also shown before and after restricting to Nicosia residents. Combining all age/sex groups ensured that there would be at least 1 cause-specific admission in at least 85% of days. The low number of daily events meant that it was not uncommon for as many as 75% of days with no admissions if age- and sex-groups would be considered separately. As about 25% and 14% of those admitted for cardiovascular and respiratory cause respectively were non-Nicosia residents, the figures reduce to an average of 2.2 and 3.5 admissions daily.

**Table 1:** Summary statistics for daily number of admissions, levels of air pollutants and meteorological factors in the 10-year period 1 Jan 1995–30 Dec 2004 (n = 3652 days).

**A. Daily number of hospital admissions for all, cardiovascular and respiratory causes**									
		Total number (% Nicosia residents)^1^	Mean	SD	Min	25%	Median	75%	Max
	
All causes		178 091	48.8	20.1	4	31	50	63	111
Cardiovascular^2^		10 896 (75%)	3.0 (2.2)	2.4 (1.9)	0 (0)	1 (1)	3 (2)	4 (3)	22 (11)
Respiratory^2^		14 827 (86%)	4.1 (3.5)	3.7 (3.1)	0 (0)	1 (1)	3 (2)	6 (5)	20 (18)
**B. Levels of air pollutants, shown separately for cold and warm months**^3^									

		Number of Days (% of total days)^4^	Mean	SD	Min	5%	Median	95%	Max
	
**Nicosia Central**									
PM_10 _24-hour average (μg/m^3^)	Cold	1553 (85.7%)	57.6	52.5	5.0	20.0	50.8	103.0	1370.6
	Warm	1664 (90.4%)	53.4	30.7	18.4	32.0	50.5	77.6	933.5
O_3 _8-hour MA max (ppb)	Cold	1514 (83.6%)	28.7	12.6	3.7	9.9	27.5	50.2	63.6
	Warm	1692 (92.0%)	44.4	10.3	7.8	24.4	46.1	58.8	71.1
**Ayia Marina**^5^									
PM_10 _24-hour average (μg/m^3^)	Cold	918 (84.5%)	25.9	28.0	6.3	9.3	19.0	62.3	553.2
	Warm	903 (81.8%)	35.7	40.5	8.1	16.0	30.9	58.9	952.4
O_3 _8-hour MA max (ppb)	Cold	1155 (80.1%)	45.7	6.8	30.2	35.1	44.6	58.4	71.0
	Warm	1247 (84.7%)	54.9	8.2	28.9	40.6	55.2	68.1	78.7
**C. Meteorological factors**									

Temperature	Cold	1812 (100%)	12.9	3.6	1.9	7.4	12.5	19.1	27.2
	Warm	1840 (100%)	25.8	3.9	11.8	19.0	26.3	31.4	35.5
Rel. Humidity		3591 (98.3%)	65.0	14.0	16.6	38.5	66.2	85.8	96.5
Dew Point		3591 (98.3%)	11.3	5.5	-7.6	2.4	10.8	20.4	24.2

**Notes: **^1 ^In the case of cause-specific admissions, information on residence was available allowing the analyses to be restricted to those resident in the Nicosia district; the same was not possible for all-cause admissions as only aggregated data were available. In parentheses, the proportion of Nicosia residents among total number of admissions. ^2 ^Summary statistics when restricting numbers to Nicosia residents are shown in parentheses. ^3 ^Cold months include Jan, Feb, Mar Apr, Nov and Dec, based on monthly average temperatures, while warm months include all the rest. ^4 ^About 10% of the 3652 days in the 10-year study period were excluded as no data were available as well as an additional 2% of days for which only less than 12 hourly measurements were recorded. ^5 ^PM_10 _and O_3 _data from the station of Ayia Marina were available only from 1999 and 1997 onwards reducing the study period to 6 and 8 years respectively.

Table 1 also shows the distribution of daily 24-hour average concentrations of PM_10 _and daily maximum 8-hour moving average of O_3 _as measured at either station as well as daily averages of the meteorological factors considered in the models. In addition to 354 (9.7%) and 387 (10.6%) days for which no PM_10 _and O_3 _were recorded at the central station respectively, an additional 81 (2.2%) and 57 (1.6%) days were excluded from the analyses as only fewer than 12 hourly measurements were available. Similarly, for the background station, around 15% of days were excluded. Daily mean levels of PM_10 _in Nicosia Central ranged from 5.0 to 1370.6 μg/m^3 ^(interquartile range: 40.0–64.1) with, as expected, slightly higher concentrations during the colder months. In the rural station, levels of PM_10 _ranged from 6.3 to 952.4 μg/m^3 ^(interquartile range: 17.0–36.3) and, in contrast to the pattern observed in the central station, appeared lower during the cold season since winter levels are mainly influenced by local sources. With the exception of extreme values thought to be the result of dust storms, levels of air pollutants in Nicosia were comparable to those seen in many southern European cities and exceeded the European standard of 75 μg/m^3 ^(at the time) between 11–57 days a year. The maximum 8-hour moving average for ozone ranged between 3.7 and 71.1 ppb (interquartile range: 26.0–48.0) in Nicosia and 28.9 and 78.7 ppb (interquartile range: 43.2–57.0) in Ayia Marina.

Table 2 presents the percentage increase in all, cardiovascular and respiratory admissions per 10 μg/m^3 ^increase in PM_10 _and 10 ppb increase in O_3 _concentrations in Nicosia Central as estimated in single-pollutant models. For a 10 μg/m^3 ^increase in same-day levels of PM_10_, there was a 0.85% (95%CI: 0.55%, 1.15%) increase in all-cause admissions. At 1.18% (95%CI: -0.01%, 2.37%) increase for every 10 μg/m^3 ^in PM_10_, a stronger association, and only just short of statistical significance, was observed with admissions for cardiovascular causes. As expected, wider confidence intervals were observed after adjusting for overdispersion. However, at 0.83% (95%CI: 0.38%, 1.28%) increase in all-cause admissions and 1.19% (95%CI: -0.10%, 2.49%) in cardiovascular admissions per 10 μg/m^3 ^increase in PM_10_, the magnitude of effects and, thus, inferences remain largely unaffected. Surprisingly, no overall association between levels of PM_10 _and respiratory admissions was observed. Furthermore, observed associations did not appear to be simply driven by days with the highest levels of PM_10_; similar, if not stronger, associations were observed when analyses were restricted to days with PM_10 _concentrations less than 100 μg/m^3^(N = 3133) and 75 μg/m^3 ^(N = 2801) – not shown in detail. Generally, no positive associations were observed with levels of PM_10 _the 2 previous days. In contrast, only effects of lagged exposure to ozone were observed. Effects appeared much stronger for cardiovascular causes, with a 2.91% (95%CI: 0.12%, 5.77%) increase for a 10 ppb increase in levels of ozone the previous day (lag 1). With the exception of a possible association in women, no overall association was observed with respiratory admissions. While it does not necessarily describe the exposure experience of the population in Nicosia, some similar associations and patterns were observed in sensitivity analyses where exposure was defined in terms of the rural station for the reduced period for which data were available. For example, a comparable 0.81% (95%CI: 0.36%, 1.27%) increase in all-cause admissions was estimated per 10 μg/m^3 ^increase in same-day levels of PM_10 _as recorded in the background station. In contrast, only weak non-significant associations were observed with cause-specific admissions. Furthermore, while generally statistically non-significant, a similar pattern of lagged effects of levels of ozone at the rural station was also observed.

**Table 2:** Percentage increase (and 95% CI) in admissions after adjusting for long- and short-term patterns as well as the effect of weather per 10 μg/m^3 ^increase in PM_10 _and 10 ppb increase in O_3 _concentrations in Nicosia Central.

**A. Per 10 μg/m**^3 ^**increase in daily 24-hour average PM**_10 _^4^				
	All admissions^2^	Cardiovascular^3^	Respiratory	Cardiovascular + Respiratory
	Lag 0^5^	Lag 0^5^	Lag 0^5^	Lag 0^5^
	
All age/sex groups	0.85 (0.55,1.15)	1.18 (-0.01,2.37)	0.10 (-0.91,1.11)	0.56 (-0.21,1.34)
Nicosia residents^1^		0.73 (-0.62,2.09)	0.25 (-0.84,1.36)	0.38 (-0.47,1.23)
				
Males	0.96 (0.54,1.39)	1.27 (-0.15,2.72)	-0.06 (-1.37,1.26)	0.63 (-0.34,1.62)
Females	0.74 (0.31,1.18)	0.99 (-1.11,3.14)	0.39 (-1.21,2.02)	0.59 (-0.68,1.87)
				
Aged <15	0.47 (-0.13,1.08)		-0.35 (-1.77,1.08)	
Aged >15	0.98 (0.63,1.33)		0.59 (-0.87,2.07)	

**B. Per 10 ppb increase in daily maximum 8-hour moving average O**_3_				

	All admissions^2^	Cardiovascular^3^	Respiratory	Cardiovascular + Respiratory
	Lag 2^5^	Lag 1^5^	Lag 2^5^	Lag 2^5^
	
All age/sex groups	0.51 (-0.16,1.18)	2.91 (0.12,5.77)	0.44 (-1.85,2.78)	0.70 (-1.05,2.49)
Nicosia residents^1^		2.48 (-0.72,5.78)	0.73 (-1.75,3.27)	0.81 (-1.13,2.80)
				
Males	0.58 (-0.35,1.52)	2.70 (-0.63,6.13)	-1.76 (-4.63,1.19)	-0.00 (-2.17,2.22)
Females	0.45 (-0.50,1.41)	3.46 (-1.53,8.70)	3.89 (0.12,7.80)	1.93 (-1.03,4.97)
				
Aged <15	1.58 (0.25,2.92)		2.27 (-0.95,5.60)	
Aged >15	0.15 (-0.62,0.92)		-1.65 (-4.89,1.70)	

Notes: ^1 ^After restricting the analyses to Nicosia residents. ^2 ^Only aggregate numbers of all-cause admissions were available, thus it was not possible to restrict numbers to Nicosia residents. ^3 ^Only includes people of adult age (15+) due to the rarity of cardiovascular events in those aged less than 15. ^4 ^Restricted to days with daily average < 150 μg/m^3^. ^5 ^Only the lag (lag 0 = same day or lag 1, 2 = previous two days) with the strongest positive association is shown.

**Figure 1:**
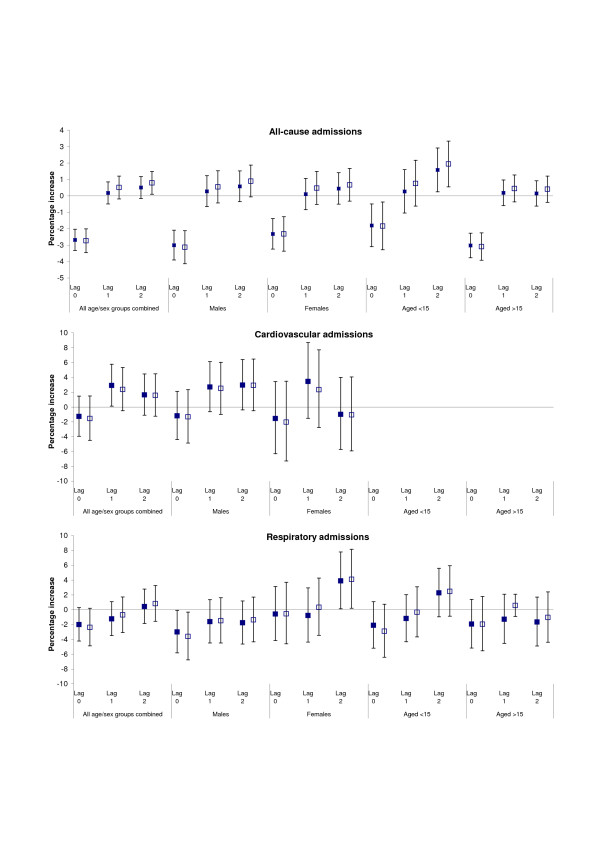
**The effect of short-term changes in ozone concentrations on hospital admissions**. Percentage increase (and 95% CI) in all, cardiovascular and respiratory admissions per 10 ppb increase in the daily maximum 8-hour moving average levels of O_3 _in Nicosia Central the same (lag 0) and two previous days (lags 1 and 2) before (*solid squares*) and after (*empty squares*) controlling for levels of PM_10 _as estimated in models adjusting for long- and short-term patterns as well as the effect of weather.

Figure 1 presents the percentage increase in admissions per 10 ppb increase in O_3 _up to two days previously before (represented with *solid *squares) and after adjusting for same-day levels of PM_10_(represented with *empty *squares). Associations with levels of ozone appeared independent of the effect of particles as adjustments either had no effect or only strengthened the observed associations. While associations with PM_10 _slightly attenuated in co-pollutant models, the conclusions were generally unchanged. Effects of ozone levels on cardiovascular admissions picked at lag 1 while for respiratory admissions were only apparent at lag 2 and appeared restricted to women and, possibly, the younger age-group. While it is true that multiple testing across sub-groups and at different lags might produce some statistically significant results, the observed patterns of a lagged effect appear consistent (even though non-significant) across causes and groups.

**Figure 2:**
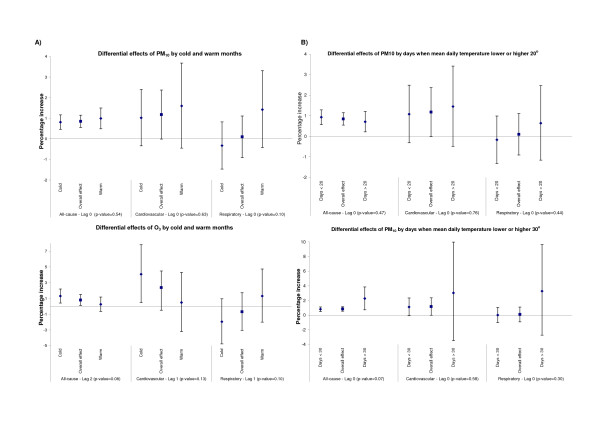
**Seasonal effects of short-term changes in concentrations of air pollutants on hospital admissions**. Percentage increase (and 95% CI) in all-cause, cardiovascular and respiratory admissions per 10 μg/m^3^increase in PM_10 _or 10 ppb increase in O_3 _(after adjusting for levels of PM_10_) by A) cold and warm months as indexed by monthly average temperature and B) cold and warm days as indexed by mean daily temperature lower or higher than 20 or 30° (shown only for PM).

While no overall association with respiratory admissions was observed, there appeared to be some pronounced differences in effect estimates by season. Table 3 shows the percentage increase in respiratory admissions (along with p-values for effect modification) across cold and warm months. While there was no effect during the colder months, an increase of 1.80% (95%CI: -0.22%, 3.85%) on admissions per 10 μg/m^3 ^increase in PM_10 _was observed during the warm season. While slight short of statistical significance, there was some suggestion of effect modification by season (p-value < 0.10). The effect, if any, seemed to be restricted to people of adult age (p-value for effect modification = 0.02) and appeared much stronger in women (p-value = 0.05). In contrast, no differential effects by season were observed in the case of all-cause and cardiovascular admissions. Figure 2 presents the estimated percentage increase in all and cause-specific admissions by (a) cold and warm months, as indexed by average monthly temperature and (b) cold and warm days as indexed by mean daily temperature lower or higher than 20 or 30°. While certainly not as pronounced (or statistically significant), the pattern of differential effects on respiratory admissions was replicated when models considered cold and warm days as indexed by the average daily temperature (instead of months). In this case, some stronger (albeit not statistically significant) effects of PM_10 _on the warmest of days (temperature > 30°) were also observed on cardiovascular admissions. Any inference, however, was constrained by the wide confidence intervals around these estimates due to the small number of days with mean daily temperature higher than 30° (N = 233). As expected, an effect of ozone on respiratory admissions also appeared somewhat stronger during the warm season. The opposite, however, was observed in the case of cardiovascular admissions, with some possibly stronger effects of ozone during the colder months.

**Table 3:** Differential effects of a 10 μg/m^3 ^increase in PM_10 _on respiratory admissions during the cold and warm season after adjusting for long- and short-term patterns as well as the effect of weather.

	Percentage increase (and 95% CI) per 10 μg/m^3 ^increase in PM_10 _^1^		
	Cold months^2^	Warm months^3^	P-value for effect modification
	
All age/sex groups	-0.33 (-1.47,0.82)	1.42 (-0.42,3.31)	0.102
Nicosia Residents^4^	-0.22 (-1.45,1.02)	1.80 (-0.22,3.85)	0.083
Males	-0.16 (-1.76,1.46)	1.10 (-1.47,3.74)	0.397
Females	-0.26 (-2.18,1.70)	3.27 (-0.00,6.65)	0.059
Aged <15	-0.31 (-2.02,1.42)	-0.59 (-3.53,2.45)	0.872
Aged 15+	0.02 (-1.76,1.83)	3.89 (1.05,6.80)	0.018

**Notes: **^1 ^Restricted to days where daily mean PM_10 _< 150 μg/m^3^.^2 3 ^Cold months include Jan, Feb, Mar Apr, Nov and Dec based on monthly average temperature; warm months include all the rest. ^4 ^After restricting the analyses to Nicosia residents.

**Figure 3:**
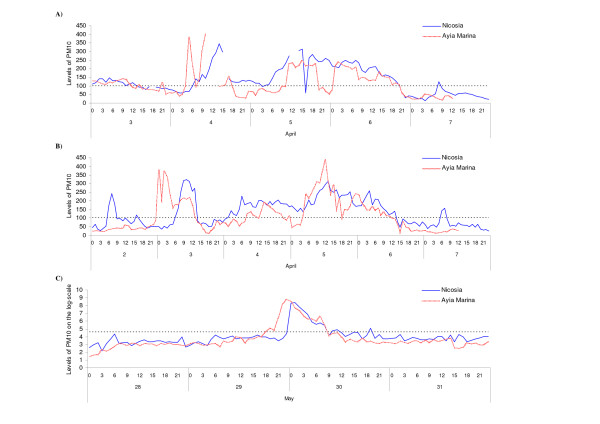
**Typical levels of PM_10 _concentrations during a dust storm**. Hourly levels of PM_10 _concentrations as recorded at Nicosia Central and Ayia Marina stations during A) a *suspected *dust storm, not confirmed by Meteorological Services records, between 4–6 April 2000, B) a dust storm confirmed by Meteorological Services records between 3–6 April 2003 and C) a confirmed dust storm with the highest recorded levels of PM_10 _(log-scale) between 29–30 May 2003.

**Figure 4:**
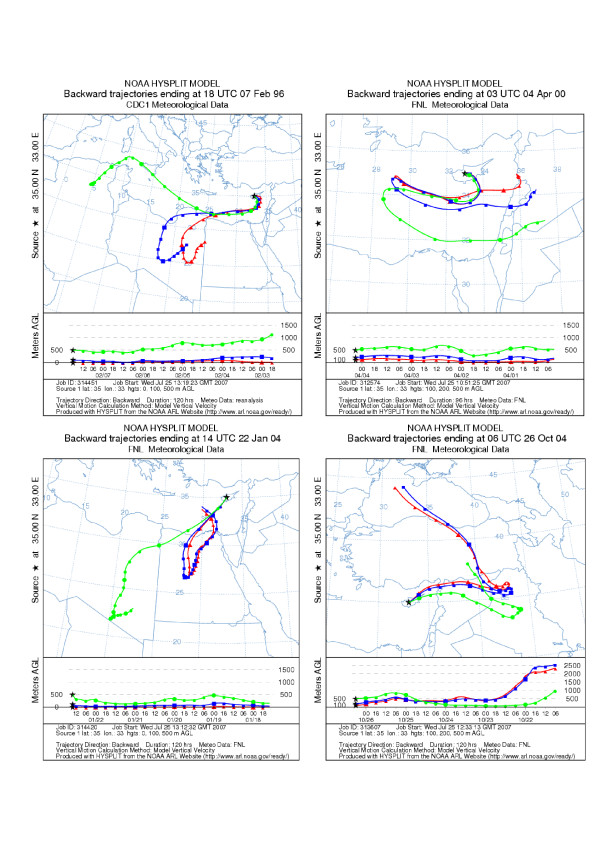
**Identification of the likely source of dust storm events**. Backwards wind trajectories ending in Nicosia on the day and about the time of the first elevated PM_10 _concentrations of the four suspected dust storm events.

A total of 63 candidate dust storm days were identified in the period under investigation (Table 4). Amongst these, a total of 45 days with uncharacteristically high levels of PM_10 _at both stations were confirmed by meteorological records. An additional 11 days identified by the Meteorological Services as dust-storm events showed relatively usual levels of PM_10_. With maximum hourly levels of PM_10 _in the background station only ranging between 22.63 and 66.6 across these days, these possibly describe events of a milder intensity. Finally, 7 days were identified that, while recorded as "Haze" by the Meteorological Services, these were nonetheless days when extreme values of PM_10 _were recorded at both stations. Since these were (a) either consecutive days (such as an event on 4–6 April 2000) or (b) they were days on either side of confirmed dust storms (such as 7 February 1996), they most likely depict "true" events overlooked by the meteorological observer. Hourly PM_10 _concentrations at either station during the "suspected" event of 4–6 April 2000 are shown in Figure 3. Both levels and patterns of PM_10 _during the three-day period appeared similar to those observed during the confirmed dust-storm event of 3–6 April 2003. Figure 4 shows 4-day backwards trajectories ending in Cyprus on the day/time of the four suspected dust storm events identifying the Sahara desert or the Arabian Peninsula as a likely source. Similar southerly (mainly in the spring) or easterly (mainly in the autumn) wind trajectories, generally consistent with previously described transport patterns [23], were observed on all other days.

**Table 4:** Calendar of confirmed and suspected dust-storm events as identified by either a meteorology observer at Larnaca airport and/or uncharacteristic levels of PM_10_.

Year	Dates	Number of days	Max number of consecutive days
1995	9 Apr*	1	1
1996	7 Feb^#^, 8 Feb, 9 Feb	3	3
1997	22 Apr*, 8 Dec	2	1
1998	16 Mar, 27 Mar, 28 Mar, 5 Jul	4	2
1999	17 Feb*, 19 Mar, 30 Mar, 7 May	4	1
2000	4 Apr^#^, 5 Apr^#^, 6 Apr^#^, 13 Apr, 18 Apr, 19 Apr, 18 Nov, 30 Dec*	8	3
2001	27 Feb*, 28 Feb*, 1 Mar, 18 Apr, 19 Apr, 22 Apr, 1 May, 13 May	8	3
2002	31 Mar*, 5 Apr, 6 Apr, 15 Apr, 1 Oct*, 19 Oct, 20 Oct, 10 Nov	8	2
2003	13 Jan, 2 Feb, 18 Feb*, 2 Mar*, 18 Mar, 19 Mar, 3 Apr, 4 Apr, 5 Apr, 6 Apr, 17 Apr*, 24 Apr, 29 May, 30 May, 11 Sept, 17 Sept	16	4
2004	16 Jan, 22 Jan^#^, 5 Mar, 27 Mar, 7 May, 10 May, 26 Oct^#^, 27 Oct^#^	9	2
**Total**		**63**	**4**

Notes: * Days which were identified as dust storms by the Meteorological services but for which levels of PM_10 _were not uncharacteristically high at either station, n = 11, possibly indicating dust storms of milder intensity ^# ^Days which were not identified as dust storms by the Meteorological Services but PM_10 _were uncharacteristically high at both stations, n = 7.

**Figure 5:**
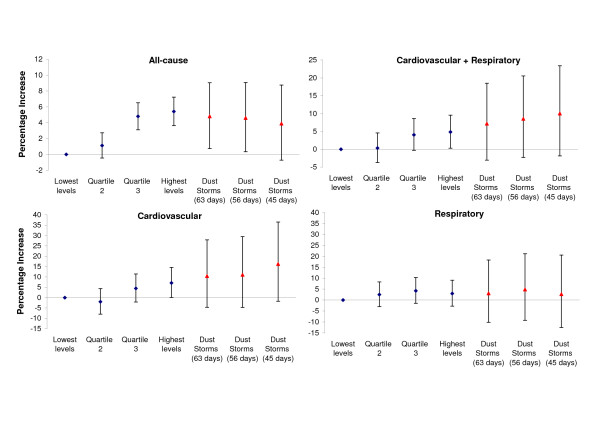
**Hospital admissions on dust storm days as compared to non-storm days**. Percentage increase (and 95% CI) in all, cardiovascular and respiratory admissions across quartiles of days with increasing levels of particulate matter (PM_10_), compared to the quarter of days with the lowest levels, after adjusting for seasonality and the confounding effect of weather. Shown separately, the estimated percentage increase in admissions on dust-storm days (n = 63 all candidate days, 56 confirmed dates irrespective of levels of PM and 45 confirmed dates with the highest levels of PM). Note: scales are not comparable across graphs.

Figure 5 plots the percentage increase in hospital admissions observed on (i) all 63 days as well as (ii) across the 56 days of confirmed events irrespective of levels of PM_10 _i.e. including storms of milder intensity and (iii) restricted to the 45 days of confirmed events with the highest levels of PM_10_. On the 63 dust-storm days, admissions were 4.8% (95%CI: 0.7%, 9.0%), 10.4% (95%CI: -4.7%, 27.9%) and 3.1% (95%CI: -10.2%, 18.3%) higher for all, cardiovascular and respiratory admissions respectively. While the observed effect achieved statistical significance only in the case of all-cause admissions, this is likely to be a result of the small number of daily events in the case of cause-specific admissions. The overall effect of dust storms on the risk of hospitalisation appeared similar in men and women, 5.7% (95%CI: 0.1%, 11.6%) and 4.0% (95%CI: -1.8%, 10.1%) respectively, or in those younger/older than 15 years of age, 5.6% (95%CI: -2.4%, 14.2%) and 4.7% (95%CI: -0.0%, 9.6%) respectively. Restricting the analyses to the 56 or 45 confirmed dust-storm days did not affect the conclusions; if anything, the effect on cardiovascular admissions appeared stronger.

For comparison purposes, figure 5 also presents the percentage increase in admissions across quartiles of non-storm days with increasing levels of PM_10 _after adjustments for seasonality and the confounding effect of weather. Stepwise increases were observed for all and cardiovascular causes, with 5.4% (95%CI: 3.6%, 7.2%) and 7.1% (95%CI 0.1%, 14.6%) increased admissions respectively in the quartile of days with the highest levels of PM_10 _(>64 μg/m^3^) compared to the quartile of days with the lowest levels (PM_10 _< 40 μg/m^3^); in both cases, p-value for linear trend < 0.01. This was not the case with respiratory admissions, where the risk of admissions rose by 2.4% (95%CI: -3.0%, 8.2%), 4.2% (95%CI: -1.5%, 10.2%) and 2.8% (95%CI: -2.8%, 8.8%) across quartiles, at least partly explaining the lack of an overall linear association. In all cases, however, the magnitude of effects on dust-storm days appeared at least comparable to the effects seen on non-storm days with the highest PM levels.

## Discussion

### Main findings

Short-term changes in PM_10 _increase the risk of same-day hospitalization for all-cause and cardiovascular causes in Nicosia hospitals. An effect on respiratory health was observed only during the warm season. In contrast to the effects seen with same-day levels of PM_10_, only lagged effects were observed with levels of ozone. These associations appeared independent of the effect of levels of PM_10 _and, perhaps, stronger for cardiovascular causes. More interestingly, there was also evidence to suggest that risk of hospitalization was higher on dust storm days. While a statistically significant association was observed only for overall admissions, in all cases, effects were at least comparable in magnitude to those seen on non-storm days with the highest levels of PM_10_, particularly for cardiovascular causes.

### Limitations

Several studies have shown that the effect of air pollution on hospitalization is stronger for certain cardio-respiratory conditions [24] and among certain sub-groups of the population, such as children, the elderly or people with a recent history [25-28]. The small number of daily events in a city the size of Nicosia provides limited statistical power to permit a finer age-, sex, or cause-specific analysis. This is, in fact, portrayed in the often low precision of the estimates and, thus, wide confidence intervals. Furthermore, multiple testing across sub-groups or different lags might produce some spurious associations. For this reason, the sub-group analyses were *a priori *restricted to gender (only)- and age (only)-specific comparisons and lagged exposure for up to 2 days. Due to lack of statistical power in a small sample, distributed lag models were not considered. Models investigating effect modification and non-linear effects across quartiles are only complimentary to the main analysis, nevertheless necessary, in the first case to highlight important differences by season and, in the case of the latter, to provide a basis of comparison (and a similar unit of measurement) between dust storm and non-storm days. The observed patterns appear not only internally consistent but, as a result of the long period of investigation (i.e. 10-years), the magnitude of estimates in a small city the size of Nicosia seem to be in agreement with those observed in large studies elsewhere.

As with all hospital data, there might be to some extent misclassification of the cause of admission, particularly in people with both respiratory and cardiovascular pathologies. However, it is unlikely that such misclassification is temporally related to levels of air pollutants and, thus, can only bias our estimates towards the null. With only one air quality station in Nicosia in operation for the full 10-year period of investigation, the analyses presented here are based on a single exposure series. Thus, replacing missing values in about 10% of days (and an additional 2–3% days purposefully excluded due to availability of less than 12 hourly measurements) was not possible. Nevertheless, the data series is generally longer than in similar time-series studies. Furthermore, similar patterns were observed when analyses were repeated for the reduced period for which data were available from the background station. Finally, identification of dust-storm days was mainly based on visibility observations and coding practices of a meteorological observer. Subjective in nature as it may be, a series of data-based criteria were used to validate the observations. It is likely that some dust storms, especially those of milder intensity, might have been missed out. However, it is unlikely that days with high levels of PM solely due to traffic sources were considered as dust-storm days. At any rate, the observed effect persisted when restricting inferences to the group of days for which meteorological observations and measurements at the three sites were in agreement.

### The effect of short-term changes in air pollutants on hospitalisation

Even though at limited power due to the small number of daily events in a city the size of Nicosia, the long period of investigation has allowed for some positive (and statistically significant) associations between levels of particulate matter and risk of hospitalization to be observed. Estimates seem consistent with the size of effects seen across several European cities, particularly for cardiovascular causes (i.e. 0.4%–1.4% per 10 μg/m^3 ^increase in PM) [29,30]. Surprisingly, other than the warm season, no overall effect was observed with respiratory admissions. While several studies have shown associations between levels of PM and respiratory mortality [6,10], there have been some inconsistent results in the case of hospital admissions [31], with some studies only showing associations in certain sub-groups [32] or for certain respiratory causes, such as COPD and asthma [33]. Furthermore, admissions during the colder months are mainly driven by viral respiratory infections. Even though models adjusted for the observed seasonal pattern with higher respiratory admissions in the colder season, it is possible that not explicitly controlling for these causes (due to lack of data) has masked a possible effect in the colder months, particularly in children (aged less than 15). It is also important to note that, in the case of respiratory admissions, a striking weakly pattern was observed with a large dip in admissions on Tuesdays (2 daily admissions on average) on either side of high volume on Mondays and Wednesdays (9 daily admissions). With the exception of the elderly (aged 65+), similar patterns were observed in all age and sex groups. While it was not clear whether this was due to reduced bed availability, there was some indication that people admitted on Monday were more likely to be discharged before Wednesday (42.5%) while those admitted on Wednesday were more likely to be kept until the following Monday (as many as 37% and only 13% for any longer). The extent to which this atypical weekly pattern has produced a discrepancy between actual need and admission and, thus, contributed to the lack of any strong association with overall respiratory admissions is not known.

No positive effects were observed with levels of PM_10 _the 2 previous days. While lagged or cumulative effects have commonly been observed between levels of PM and mortality in several studies [34], it is not uncommon for the strongest associations to be observed with same-day levels in the case of hospital admissions [12,35]. Perhaps, even more so in the case of Cypriot cities where, with no referral system, direct access to specialist health care may be considerably easier than in larger European or US cities. A number of person-based studies have now used average levels of air pollutants the 24 hours preceding an event (rather than same calendar day which would include hours after as well as before the event) and, at least amongst susceptible populations, have shown effects in as little as 6 hours prior to an arrhythmia and even 2 hours prior to a myocardial infarction [36-39].

In contrast to the same-day association with levels of PM, only effects of lagged exposure to ozone were observed. Negative associations with same-day levels of ozone have previously been observed in several of the 23 APHEA cities, an effect which persisted even in the summer period in at least 4 of the cities, namely Rome, Paris, Tel Aviv and Valencia [40]. While explanations for this are not clear, it is thought to be a product of the relationship between ozone (a secondary pollutant produced by photochemical reactions) and traffic-related primary pollutants such as nitrogen oxides (NO) and volatile organic compounds (VOC) which can reduce ozone concentrations at least at the local scale. Thus, high ozone concentrations in stations located in central parts of cities, such as in this case, may reflect low levels of local traffic pollutants or good dispersion conditions, which can lead to negative associations with health indicators, at least in the short-run, whereas it is common for stronger associations with ozone to be subsequently observed at lagged intervals. A similar pattern of negative same-day associations, albeit much weaker, was observed with levels of ozone as recorded at the rural station, not influenced by local pollution. Finally, while, there have generally been some inconsistent findings for the association between cardiovascular morbidity and levels of ozone, with some reporting positive [41], no or even negative associations [42], effects of a similar magnitude have been previously reported between levels of ozone two days before admission for cardiovascular causes in London [43]. In general, neither the fact that stronger associations with lagged rather than concurrent exposure nor that stronger effects for cardiovascular than respiratory admissions were observed seem inconsistent with previous findings [43-45].

### Effect modification by season

Seasonal differential effects of changes in levels of air pollution on the risk of hospitalization have been previously reported, particularly with respects to stronger effects of PM on respiratory health during the warm season such as those observed here [32,43,46]. Other studies, however, have found little evidence of differential effects by season [47,48]. It is uncertain whether such differential effects carry biological plausibility, i.e. a synergistic effect of air pollution and temperature amplifying people's response to lower levels of air pollutants than normally, or it simply, reflects an increased proportion of time spent outdoors, and thus higher exposure, on warm days [49]. In APHEA, for instance, the stronger effects of PM_10 _on total mortality observed in the warmer than colder cities persisted when latitude was used instead of actual temperature, thus, it was proposed that ambient measurements in warmer places may represent the average population exposure more accurately than in colder places where people do not spend as much time outdoors [21]. Here, other than some weak evidence of strongest effects on cardio-respiratory morbidity on the warmest days (>30°), generally, more pronounced differential effects were observed across cold-warm months rather than across cold-warm days (based on daily mean temperature).

In the light of evidence of ozone effects on mortality restricted to the warm season across 23 APHEA cities, the stronger association between cardiovascular admissions and ozone during the colder season observed here appears surprising [40]. However, some similar patterns have been observed elsewhere, particularly amongst the elderly [50]; possible explanations for this pattern are unclear and it might simply be a chance finding. Nonetheless, in a recent cross-sectional study in Korea, it was reported that measures of blood pressure, a risk factor for cardiovascular disease, were significantly associated with PM_10 _levels only in the warm season while the reverse was true for O_3_, with associations only during the cold season [51].

### The effect of dust storms

Long-range transport of Saharan dust across the Mediterranean into southern Europe [52,53], and less frequently as far north as the British Isles [54], is well established. Consistent with a recent examination of these events [23], our analysis also suggests that there has been a rise in their frequency, at least half of which seem to occur during the spring months and can last for as many as four consecutive days. Unlike the mineral and chemical composition as well as transport patterns of Saharan dust, the possible health effects of these natural events have not been extensively studied. There is some modest evidence from Taiwan and Korea of adverse effects of wind-blown dust from the Mongolia/China desert on both cardiovascular and respiratory health, some times lasting for up 3 days after the event [55-57]; reported associations were, however, not always statistically significant.

In contrast, a study of 17 episodes with a high concentration of coarse (crustal-derived) but not fine particles during a six-year period in Spokane, Washington, revealed no evidence of an increased risk of death on dust days [14]. Similarly, an investigation into an unusual event of transported dust over the Atlantic to Greater Vancouver, Canada, has shown no effect on hospital admissions [58]. Some studies have, in fact, reported strengthening of the observed health effects when windblown dust days were excluded from the analyses [59] or reduced health effects on windy days [60], in both cases suggestive that crustal-derived particles are more benign that those from anthropogenic processes. In our analyses, we have not observed an attenuation of effects by including dust-storm days. On the contrary, we found evidence of increased admissions on dust-storm days of similar magnitude to the effects seen on non-storm days with the highest concentrations. Of course, the possibility of air-borne dust containing particles of anthropogenic sources can not be excluded since mega-cities, such as Cairo, are commonly on the path of these events. Alternatively, some support for the adverse effects of particles due to natural sources observed here comes from a recent study of the biological content in dust transferred to Haifa, Israel during similar events in 2004–2005 that revealed both an increase in the concentration of airborne microorganisms and a change in the usual content of fungal population in PM samples, both which are thought to affect human health [19]. Similar findings have been reported from aeromicrobiological analyses of samples on the Turkish coastal town of Erdemli [61].

## Conclusion

We observed an increased risk of hospitalization at elevated levels of particulate matter and ozone generally consistent with the magnitude seen across several European cities. Interestingly, we also observed an increased risk of hospitalization on dust storm days, particularly for cardiovascular causes. While inference from these associations is limited due to the small number of dust storm days in the study period, these effects did not appear to be an artifact of including days of high traffic pollution. While these represent non-preventable events, with a magnitude of effects at least comparable to those on days with the highest levels of PM_10 _from traffic sources, such events may merit special health warnings directed to the most vulnerable population groups.

## List of abbreviations

μg/m^3^: Micrograms per cubic metre; AIC: Akaike information criterion; APHEA: The Air Pollution and Health: a European Approach Project; CI: Confidence Interval; ICD: International Classification of Diseases; HYSPLIT: Hybrid Single-Particle Lagrangian Integrated Trajectory; GAM: Generalized Additive Models; NMMAPS: The National Morbidity, Mortality and Air Pollution Study; NOAA: National Oceanic and Atmospheric Administration; O_3_: Ozone; PM_10_: Particulate matter with aerodynamic diameter less than 10 microns; ppb: parts per billion; SD: Standard deviation; TEOM: Tapered Element Oscilling Micro-balance; UBRE: Unbiased Risk Estimator.

## Competing interests

The authors declare that they have no competing interests.

## Authors' contributions

PY, PD and PK conceived and secured funding for the study from the Cyprus Ministry of Health. PY, NM and OK collected the data. NM compiled the data and performed the statistical analyses. PK, JS and DD provided guidance and statistical advice. All authors assisted in the interpretation of results. NM wrote the first draft of this paper. All authors contributed towards the final version. All authors read and approved the final manuscript.
